# Mechanical Complications in Adult Spine Deformity Surgery: Retrospective Evaluation of Incidence, Clinical Impact and Risk Factors in a Single-Center Large Series

**DOI:** 10.3390/jcm10091811

**Published:** 2021-04-21

**Authors:** Giuseppe Barone, Fabrizio Giudici, Nicolò Martinelli, Domenico Ravier, Stefano Muzzi, Leone Minoia, Antonino Zagra, Laura Scaramuzzo

**Affiliations:** IRCCS Istituto Ortopedico Galeazzi, 20100 Milan, Italy; giuseppebarone.gale@gmail.com (G.B.); fabrizio.giudici@grupposandonato.it (F.G.); domenicoravier@gmail.com (D.R.); stefano.muzzi@unimi.it (S.M.); leone.minoia@grupposandonato.it (L.M.); antonino.zagra@grupposandonato.it (A.Z.); scaramuzzolaura@gmail.com (L.S.)

**Keywords:** adult spine deformity, complications, clinical outcomes

## Abstract

The advancement of deformity-specific implants and surgical techniques has improved the surgical treatment of Adult Spine Deformity (ASD), allowing surgeons to treat more complex deformities. Simultaneously, high rates of medical and surgical complications have been reported. The aim of this study is to describe the risk factors, the rate and the clinical impact of mechanical complications in ASD surgery. A retrospective review of a large, single-center database of consecutive ASD patients was conducted. Inclusion criteria were as follows: Cobb coronal curve > 20° or alteration of at least one of sagittal vertical axis (SVA > 40 mm), thoracic kyphosis (TK > 60°), pelvic tilt (PT > 20°) and pelvic incidence minus lumbar lordosis mismatch (PI-LL > 10°), at least four levels of posterior instrumented fusion and 2-year follow-up. At the baseline and at each follow-up end point, the authors collected clinical and radiographic outcomes and recorded any mechanical complications that occurred. One hundred and two patients were enrolled. Clinical outcomes significantly were improved at the last follow-up (mean 40.9 months). Postoperative mechanical complications occurred in 15 patients (14.7%); proximal junctional disease was the most common complication (60%) and the revision rate was 53.3%. Patients who experienced mechanical complications were older (61.2 vs. 54.8 years, *p* = 0.04); they had also a higher rate of pelvic fusion and posterior-only approach, a lower LL (−37.9 vs. −46.2, *p* = 0.02) and a higher PT (26.3 vs. 19.8, *p* = 0.009), TK (41.8 vs. 35.7, *p* = 0.05), PI–LL (12.9 vs. 5.4, *p* = 0.03) and Global Alignment and Proportion score (6.9 vs. 4.3, *p* = 0.01). This study showed a significant improvement in pain and disability after ASD surgery. Regarding the risk of developing a mechanical complication, not only postoperative radiographic parameters affected the risk but also patient age and surgical features.

## 1. Introduction

Adult spine deformity (ASD) includes a complex group of pathologies that covers a broad range of radiographic patterns with various clinical presentations in skeletally mature patients [1]. ASD can occur as a degenerative process of aging, as a secondary progression to juvenile/adolescent scoliosis or kyphosis and can be a result of prior surgery.

A recent study by Schwab et al. showed a prevalence of 68% of ASD in patients older than 60 years [2]. ASD can be asymptomatic but it is more often progressive, leading to severe axial back pain, gait instability and neurological impairments due to compression of neural elements. Moreover, different studies found that compensatory mechanisms reducing sagittal malalignment were associated with greater disability, pain and energy expenditure [3].

Multiple studies have also shown a potential significant improvement in pain and disability with surgery for ASD, based on health-related quality of life (HRQOL) scores [4,5,6,7,8]. Nowadays, the advancement of deformity-specific implants and surgical techniques has improved the surgical treatment of ASD, allowing surgeons to treat more complex deformities. At the same time, high rates of medical and surgical complications have been reported, ranging from 10 to 78% [8,9,10].

Surgical complications can be differentiated into mechanical (e.g., proximal/distal junctional failure, rod breakage, implant pullout) and non-mechanical (e.g., dural tears, neurological deficits, infections).

Despite numerous ASD surgery studies, the literature is sparse regarding the impact of mechanical complications. The aim of this study is to describe the risk factors and the rate of mechanical complications, as well as the clinical outcome at the last follow-up, in a single-center, large series of patients who underwent ASD surgery.

## 2. Materials and Methods

The authors reviewed a prospectively collected database which recorded demographic, surgical and radiographic data on consecutive patients who underwent surgical treatment for ASD in a single spine surgery division, from September 2010 to July 2016. Four expert surgeons were involved in surgical procedures. The study was conducted according to the Declaration of Helsinki and Good Clinical Practice and was approved by the local ethical committee.

Inclusion criteria were patients older than 25 years, long posterior or combined instrumented fusion (at least 4 instrumented vertebrae), at least 2-year follow-up, diagnosis of spinal deformity defined by one or more of these preoperative radiographic findings: coronal Cobb angle ≥ 20°, sagittal vertical axis ≥ 40 mm, thoracic kyphosis ≥ 60°, pelvic tilt ≥ 20° and pelvic incidence minus lumbar lordosis mismatch ≥ 10°, according to SRS-Schwab classification [1]. The authors excluded patients with an incomplete dataset and previous corrective spinal surgery for their deformity.

Standard demographic data, including age, gender, height, weight and body mass index (BMI), were collected at admission. The following data were recorded for each intervention: surgical approach (only posterior or combined lateral trans-psoas and posterior approach), number of fused vertebrae, proximal and distal limits of the instrumentation, iliac extension of the construct, application of interbody cages (through posterior or lateral trans-psoas approach). Radiographic data were measured by a single expert examiner on antero-posterior and lateral plain full standing X-rays, preoperatively and early postoperatively (1 month), using validated software (Sectra Workstation; Sectra AB, Linkoping, Sweden). Several variables were analyzed: coronal Cobb angle of the main curve, sagittal vertical axis (SVA), lumbar lordosis (LL-L1-S1), sacral slope (SS), pelvic tilt (PT), pelvic incidence (PI), PI-LL mismatch, global tilt (GT) and thoracic kyphosis (TK-T4-T12).

For each patient, the Global Alignment and Proportion (GAP) score was obtained in order to analyze the global spinopelvic alignment and to predict the risk of mechanical complications. This score was acquired measuring PI, SS, L1-S1 lordosis, L4-S1 lordosis and global tilt on early postoperative X-rays, as described by Yilgor et al. [11]. We used an automated Microsoft Excel spreadsheet to simplify calculation.

At the baseline and at each follow-up end point (early, 4-month, 12-month and every year postoperatively), the authors collected clinical outcomes and any mechanical complications through patient clinical visits and X-rays.

Clinical outcome was assessed using a visual analog scale (VAS) and the Oswestry Disability Index (ODI) as health-related quality of life score (HRQOL).

Mechanical complications were defined as proximal junctional kyphosis (PJK—≥10° increase in kyphosis between the upper instrumented vertebra—UIV—and two vertebrae above UIV, between early postop and follow-up X-rays), proximal junctional failure (PJF—fracture of UIV or one vertebra above UIV, pullout of instrumentation at UIV and/or sagittal subluxation), distal junctional kyphosis or failure (DJK or DJF—≥10° postoperative increase in kyphosis between lower instrumented vertebra—LIV—and one vertebra below LIV and/or pullout of instrumentation at LIV) single or double rod breakage and other implant-related complications, such as loosening or breakage of the screws and dislodgment of interbody devices or hooks [12]. Patients were divided into two groups: Group A, in which no complication occurred, and Group B, in which patients suffered at least one mechanical complication, early (within 6 month after surgery) or delayed.

### Statistical Analysis

Statistical analysis was performed using IBM SPSS (SPSS Statistics V20; SPSS, Inc., an IBM Company, Chicago, IL, USA) and results were expressed using means and standard deviation. The data were assessed using the Shapiro–Wilk test for normality. For categorical variables, Pearson chi-square tests were used to compare distributions between two groups. For numerical variables, statistical comparison was performed using Student t-tests (normal distribution) or Mann–Whitney U-tests (not normal distribution). Statistical analyses were 2-sided and the level of significance for each statistical test was set at *p* < 0.05.

## 3. Results

**Figure 1:**
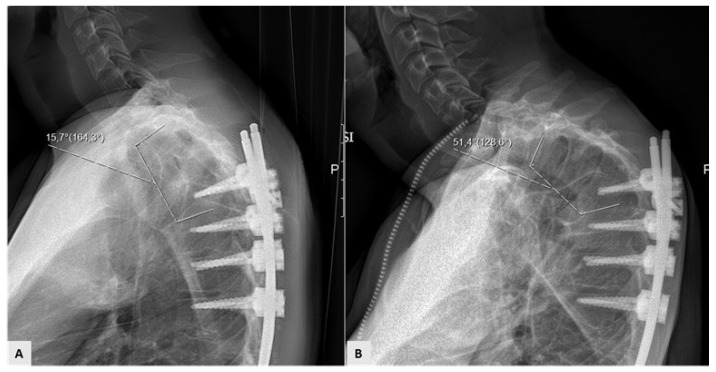
Example of proximal junctional kyphosis; (**A**) early postoperative proximal junctional angle; (**B**) 4-month increase (>10°) in kyphosis between the upper instrumented vertebra—UIV—and two vertebrae above UIV.

**Figure 2:**
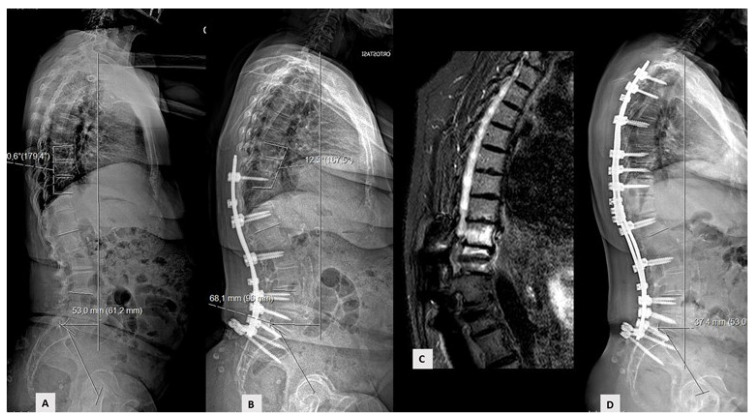
Example of proximal junctional failure. (**A**) Preoperative sagittal X-ray; (**B**,**C**) 12-month postoperative X-ray and magnetic resonance revealed fracture and sagittal subluxation one vertebra above upper instrumented vertebra; (**D**) X-ray after revision surgery.

Thirty-two patients out of 134 patients recorded in the database were excluded for incomplete datasets. One hundred and two patients met the inclusion criteria and were enrolled. Mean follow-up was 40.9 months (range 24–96); 87 patients had no mechanical complications (Group A—85.3%). Fifteen patients (Group B—14.7%) experienced mechanical complications, including 5 PJK (Figure 1), 4 PJF (Figure 2), 3 rod breakage, 2 DJF and 1 screw loosening.

Complications occurred within 6 months after surgery in five patients (33.3%). Eight patients (53.3%) out of the 15 patients who experienced a mechanical complication underwent revision surgery (Table 1).

All patients were instrumented with 5.5-mm-diameter cobalt-chrome rods and pedicle screws; posterior arthrodesis was completed by using autologous and synthetic morcellized bone graft. Twenty-seven patients (26.5%) underwent combined surgery (lateral trans-psoas approach and posterior open approach). Minor multilevel osteotomies (bilateral facetectomy, Smith–Petersen osteotomy, Ponte osteotomy) were performed when sagittal correction of the deformity was required. No patients underwent an anterior approach or three-column osteotomy. Thirty-five patients underwent circumferential fusion while 67 patients had posterior-only fusion. Interbody cage was used in case of local instability or when an increase in segmental lordosis was necessary. Comparison between these groups did not lead to a statistically significant difference (*p* > 0.05).

At the last follow-up, both groups experienced improvement of the clinical outcomes. The differences in the postoperative amount of clinical improvement between the two groups were statistically significant (Table 2).

The results of univariate analysis of the impact of patient demographic, surgical and radiographic differences on the incidence of mechanical complications are shown in Table 3 and Table 4. No statistically significant differences were detected between Group A and Group B regarding average height, weight, BMI and gender. Patients who experienced mechanical complications were older at the time of surgery (61.2 years vs. 54.8 years, *p* = 0.04). Moreover, patients in Group B had the largest number of instrumented vertebrae, a higher rate of long thoracic fusions with an upper instrumented vertebra between T1 and T5 and more frequent pelvic extension of the arthrodesis area. Patients in Group A showed a higher rate of combined trans-psoas and posterior approach (Table 3).

Preoperatively, no differences were detected in the coronal Cobb angle of the main curve, sagittal vertical axis or pelvic tilt between the two groups. Patients in Group B had a lower lumbar lordosis and SS, larger PI-LL mismatch, higher global tilt and thoracic kyphosis. However, these differences were not statistically significant, indicating good homogeneity between the two groups at baseline (Table 4).

Postoperatively, at early radiographic follow-up (1 month), patients in Group B had a higher SVA, PT (26.3° vs. 19.8°, *p* = 0.009), PI-LL mismatch (12.9° vs. 5.4°, *p* = 0.03), GT and TK (41.8° vs. 35.7°, *p* = 0.05). Furthermore, patients who experienced mechanical complications showed a lower postoperative lumbar lordosis (−37.9° vs. −46.2°, *p* = 0.02). No significant difference was reported in the postoperative coronal Cobb angle of the main curve between the two groups (Table 4).

Patients who experienced postoperative mechanical complications had a statistically significantly higher GAP score (6.9 vs. 4.3, *p* = 0.01) (Table 5). Patients with a proportioned spinopelvic state according to the GAP score (0–2 points) had a mechanical complication rate of 7.9%, while those with a moderately (GAP: 3–6 points) or severely (GAP ≥ 7 points) disproportioned state had mechanical complication rates of 14.2% and 22.2%, respectively.

Table 6 shows demographic data and different parameters of patients with mechanical complications (Table 6).

## 4. Discussion

In this study, we assessed mechanical complications that occurred in a large, single-center, consecutive series of patients who underwent surgical treatment for ASD. We identified potential predictor factors of mechanical complications among patient demographic parameters and preoperative and postoperative radiographic findings. Furthermore, we analyzed the clinical impact of these complications on patients’ quality of life. Multicenter studies showed wide variability in complications among different sites and different surgeons in the same institution (10–78%). This could be explained by the high heterogeneity of etiologies, definitions, population baseline features, follow-up and surgical techniques [8,9,10]. In our series, the incidence of mechanical complications was 14.7%, with an average follow-up of 40.9 months; more than one-half of these patients required revision surgery.

Regarding clinical outcomes, both groups experienced a significant improvement over time. Obviously, the differences in postoperative amount of clinical improvement between the two groups were statistically significant.

Proximal junctional disease (PJK and PJF) represented the main cause of mechanical failure in our series (60%). This result was similar to those reported in the recent literature. Proximal junctional kyphosis is a radiographic finding defined as an increase in kyphosis of ≥10°, between the UIV and two vertebrae above the UIV, between early postop and follow-up X-rays. Proximal junctional failure, instead, refers to the fracture of the UIV or one vertebra above the UIV, pullout of instrumentation at UIV and/or sagittal subluxation [11]. These phenomena are probably due to the increased junctional stress, often associated with long posterior spinal fusion, patient’s age, pre-existing low bone mineral density, preoperative hyper-kyphosis, inappropriate sagittal alignment and surgery-related factors (e.g., extensive para-spinal muscle dissection at the UIV, disruption of the supraspinous and interspinous ligaments, improper end-vertebra selection, facet violation, pelvic fixation, magnitude of correction) [12,13,14,15,16]. Several prevention strategies were suggested to reduce the incidence of PJK and PJF, such as UIV + 1 cement augmentation, use of spinal hooks, sublaminar band placement at UIV + 1. Recent studies seem to confirm that hooks can decrease the risk for PJK as they provide a more dynamic construct and are associated with lower incidence of facet violation [17,18].

PJK has been previously associated with choice of UIV level. However, despite a better knowledge of the expected outcomes after ASD surgery according to patient metrics, there is no clear evidence to which radiographic and clinical factors should be taken into account to choose between fusing the lower-thoracic (LT) or upper-thoracic (UT) spine. In our study, five patients developed PJK: in three patients, UIV was in the UT spine (T5 or upper vertebra), while, in two patients, the UIV was in the LT spine (T9 or lower). However, the difference between UT and LT for PJK rates was not statistically significant (*p* > 0.05). The lack of statistical significance for PJK may thus be related to the limited power for the detection of the true differences in this relatively less common complication.

Single- or double-rod breakage accounted for 20% of our implant-related complications. This phenomenon has been associated with overcorrection of deformity because of overload on implant (especially when spine osteotomies are performed), poor sagittal alignment restoration, non-unions and mechanical instability. In order to avoid this complication, some options have been proposed, such as variation in rod material (cobalt-chrome rods have higher stiffness and fatigue life with respect to titanium alloy rods) and configuration, multi-rod constructs and local bone morphogenetic protein application [10,13,15,19,20,21,22,23].

In our series, the results of statistical analysis revealed that patient’s age was the only potential risk factor for mechanical complications. Patients who experienced a mechanical complication were 6.4 years older. The impact of age on the outcome of patients treated for ASD is well established [6]. The mean age (55.7 years) of our series may explain the lower complication rate in comparison to the literature. However, Smith et al. showed that older patients could have similar results, in terms of disability and health status, to younger patients [6].

The complication rate of older patients may be due to osteoporosis, age-related muscle–skeletal and neurosensory changes, gait and balance disorders or other medical comorbidities. Interestingly, there were no differences regarding BMI, height or weight. This finding contrasts with other series in the literature in which BMI or weight are associated with higher risk of mechanical complications [10,13].

Regarding surgical features, the number of fused vertebrae and the rate of interbody cage application were not statistically significant between the two groups. Conversely, patients who had an upper instrumented vertebra between T1 and T5 or iliac extension of the arthrodesis area showed a trend toward having a mechanical complication; these findings could be explained considering that more complex deformities are more likely to have mechanical complications. Furthermore, patients in Group A showed a trend toward having a higher rate of combined trans-psoas and posterior approach. This could be explained by the biomechanical advantage of an improved anterior column support given by the application of a large lordotic interbody cage.

The prevalence of mechanical complications in our patients (14.7%) was significantly lower than most reported series. This could be due to the heterogeneity of age, radiographic features of deformity and surgical techniques [12,13,14,20]. Indeed, we observed that the main difference between our study cohort and other series was that we never used complex three-column osteotomies (3-CO), widely performed for sagittal plane correction in ASD. Maier et al. reported 20.9% of revision surgery within 1 year, only for mechanical complications, in 335 patients who underwent 3-CO for ASD correction [20]. In a multicenter, consecutive series with a 2-year follow-up, the rate of implant-related complications in 82 3-CO patients was 40.2% [10]. Soroceanu et al., in a large series of 245 ASD patients, identified that nearly one-third (31.7%) of patients undergoing surgery experienced a radiographic or implant-related complication and that more than one-half of these patients required reoperation within 2 years of surgery [13].

As mentioned previously, no patients in our series underwent three-column osteotomy; when we needed to treat sagittal plane deformities, minor posterior column osteotomies or lordotic interbody cage application were performed. This could be a crucial factor associated with our relatively low incidence of mechanical complications. Probably for the same reason, our patients did not often achieve a significant postoperative recovery of LL, PT or ideal sagittal alignment. However, this does not appear to affect the clinical outcomes, as the patients showed a statistically significant improvement in mean postoperative VAS and ODI score. Three-column osteotomies could have reached better radiographic alignment in severe cases of ASD. The increase in the complication rate in such cases should be assessed in further studies.

Regarding radiographic features, recent research showed that sagittal spinopelvic alignment among patients with ASD plays a critical role in pain and disability and it is a primary determinant of health-related quality of life scores [2,3,4,5,21] Our series confirms that the coronal Cobb angle of the main curve does not seem to be related to an increased risk of mechanical complications. Instead, patients who experienced a mechanical complication significantly differed in postoperative LL, PT, PI-LL mismatch and TK.

In order to define the global sagittal pattern of patients with ASD, Yilgor et al., on behalf of the European Spine Study Group, proposed a new method of analyzing spinopelvic alignment to predict mechanical complications [11]. The authors developed a score (Global Alignment and Proportion—GAP score) that provides pelvic incidence-based “ideal values” of sagittal alignment for any patient. These values include the relative pelvic version, relative lumbar lordosis, lordosis distribution index, relative spinopelvic alignment and an age factor [11]. The GAP score can range from 0 to 13 points, in proportion to the difference between postoperative measured parameters and the ideal ones. We applied this score at our study cohort to verify its reliability. Patients who experienced postoperative mechanical complications had a statistically significantly higher GAP score (6.9 vs. 4.3, *p* = 0.01), suggesting that the GAP score could be considered a useful tool to predict mechanical complications with a good reliability.

The strengths of this study are the large, consecutive cohort of homogenous patients, the uniform surgical technique and the duration of the follow-up interval. The limitations are the retrospective design of the study and the limited number of patients who developed a mechanical complication, which reduces the power of our findings. Furthermore, although age was found as a possible risk factor, osteoporosis, sarcopenia and neurocognitive factors related to aging were not directly evaluated.

## Figures and Tables

**Table 1:** Mechanical complications.

Complication Type	*n*	%
Proximal junctional kyphosis	5	33.3
Proximal junctional failure	4	26.7
Distal junctional failure	2	13.3
Rod breakage	3	20
Screws loosening	1	6.7
Early (<6 months)	5	33.3
Delayed (>6 months)	10	66.7
Revision	8	53.3

**Table 2:** Pre- and postoperative clinical findings; mean [standard deviation].

	All Patients	Group A	Group B	*p*
VAS pre	7.4	7.3 [2.1]	7.8 [1.3]	=0.38
VAS post	3.2	2.7 [2.2]	5.5 [1.8]	<0.01
*p*	<0.01	<0.01	=0.03	
ODI pre	44.6	44.2 [14.5]	46.3 [11.5]	0.6
ODI post	19.5	15.5 [13.7]	39.7 [10.2]	<0.01
*p*	<0.01	<0.01	=0.17	
∆VAS	4.2	4.6 [2.1]	2.3 [1.8]	=0.01
∆ODI	25.1	28.7 [15.9]	6.6 [11.5]	<0.01

ODI, Oswestry Disability Index; VAS, visual analogue scale; ∆VAS/ODI, pre–postoperative difference.

**Table 3:** Demographic and surgical data; mean [standard deviation] or (% of patients).

	All Patients	Group A	Group B	*p*
Patients, *n*	102	87	15	-
Age, y	55.7	54.8 (10.9)	61.2 (11.1)	0.04
F/M	91/11	76/11	15/0	0.14
Height, cm	162.1	162.3 (7.4)	161.2 (7.6)	0.62
Weight, Kg	64.5	64.7 (10.1)	63.1 (8.8)	0.53
BMI	24.5	24.6 (3.4)	24.3 (3.8)	0.8
Follow-up, m	40.9	41.2 (18)	39.6 (18.5)	0.75
Instrumented vertebrae, *n*	10.1	9.8 (2.9)	11.2 (3.2)	0.1
Iliac extension, *n*	7 (6.8)	4 (4.6)	3 (20)	0.02
UIV at T1–T5, *n*	41(40.2)	32 (36.8)	9 (60)	0.09
Cage application, *n*	35 (34.3)	32 (36.8)	3 (20)	0.2
Combined Approach, *n*	27 (26.5)	26 (29.8)	1 (6.7)	0.06

BMI, body mass index; UIV, upper instrumented vertebra.

**Table 4:** Preoperative radiographic parameters; mean [standard deviation].

	All Patients	Group A	Group B	*p*
Coronal Cobb, °	42.9	43.3 [18.5]	41.1 [21.6]	0.67
SVA, mm	39.2	38.4 [30.6]	42.3 [37.2]	0.68
LL, °	−41.5	−42.3 [17.5]	−37.9 [17.7]	0.38
SS, °	30.1	30.7 [10.7]	26.5 [13]	0.17
PT, °	21.1	20.6 [9.9]	23.7 [11.5]	0.27
PI, °	51.4	51.5 [10.5]	50.9 [12.9]	0.87
PI-LL, °	9.8	9.2 [16.6]	13.1 [17.4]	0.41
GT, °	22.8	21.8 [13.8]	27.8 [14.9]	0.12
TK, °	33.3	32.3 [14.1]	38.8 [14.1]	0.11
Postoperative radiographic parameters; mean [standard deviation]				
Coronal Cobb, °	19	19.5 [13.3]	16.4 [12.8]	0.41
SVA, mm	27.2	25.6 [21.6]	35.6 [25.9]	0.11
LL, °	−44.8	−46.2 [13.2]	−37.9 [12.6]	0.02
SS, °	30	31.2 [9.9]	24.1 [9.7]	0.01
PT, °	20.8	19.8 [8.7]	26.3 [8.5]	0.009
PI, °	51.4	51.6 [10.7]	50.8 [13.3]	0.81
PI−LL, °	6.6	5.4 [11.9]	12.9 [12.8]	0.03
GT, °	22.9	21.7 [10.3]	29 [12.1]	0.17
TK, °	36.7	35.7 [10.9]	41.8 [10.1]	0.05

GT, global tilt; LL, lumbar lordosis; PI, pelvic incidence; PT, pelvic tilt; SS, sacral slope; SVA, sagittal vertical axis; TK, thoracic kyphosis.

**Table 5:** Global Alignment and Proportion (GAP) score distribution.

	GAP Score 0–2	GAP Score 3–6	GS ≥ 7	
Patients, *n*	38	28	36	
Complications, *n*	3 (7.9%)	4 (14.2%)	8 (22.2%)	
	All patients	Group A	Group B	*p*
GAP Score, mean [SD]	4.7	4.3 [3.5]	6.9 [4.1]	0.01

**Table 6:** Details for the 15 patients with mechanical complications. BMI, body mass index; DJF, distal junctional failure; PJF, proximal junctional failure; PJK, proximal junctional kyphosis; GAP score, Global Alignment and Proportion score; UIV, upper instrumented vertebra.

Patient	Age at Intervention (Years)	Gender	BMI	Complication Type	GAP Score	UIV	Instrumented Vertebrae, *n*
1	60	F	33.3	Rod Breakage	6	T10	7
2	51	F	20.8	Screws Loosening	4	T10	7
3	63	F	22.7	DJF	10	T5	13
4	62	F	23.5	DJF	5	T11	7
5	41	F	27.9	Rod Breakage	12	T5	13
6	61	F	23	Rod Breakage	6	T11	8
7	60	F	23	PJF	2	T8	9
8	62	F	22.3	PJK	8	T4	13
9	72	F	26.7	PJK	7	T11	8
10	68	F	21.5	PJK	7	T4	15
11	35	F	19.8	PJF	0	T4	11
12	73	F	30.9	PJF	13	T4	15
13	75	F	25.4	PJF	13	T4	15
14	66	F	21.4	PJK	9	T4	15
15	55	F	23.1	PJK	1	T5	12

## Data Availability

The data that support the findings of this study are available from the first author upon reasonable request.
